# Morphology shapes community dynamics in early animal ecosystems

**DOI:** 10.1038/s41559-024-02422-8

**Published:** 2024-06-12

**Authors:** Nile P. Stephenson, Katie M. Delahooke, Nicole Barnes, Benjamin W. T. Rideout, Charlotte G. Kenchington, Andrea Manica, Emily G. Mitchell

**Affiliations:** 1 Department of Zoology, University of Cambridge, Cambridge, UK; 2 University Museum of Zoology, University of Cambridge, Cambridge, UK; 3 Department of Earth Sciences, University of Cambridge, Cambridge, UK; 4 Independent Researchers, https://www.nature.com/natecolevol/

**Keywords:** Community ecology, Palaeontology, Palaeoecology

## Abstract

The driving forces behind the evolution of early metazoans are not well understood, but key insights into their ecology and evolution can be gained through ecological analyses of the in situ, sessile communities of the Avalon assemblage in the Ediacaran (~565 million years ago). Community structure in the Avalon is thought to be underpinned by epifaunal tiering and ecological succession, which we investigate in this study in 18 Avalon communities. Here we found that Avalon communities form four distinctive Community Types irrespective of succession processes, which are instead based on the dominance of morphologically distinct taxa, and that tiering is prevalent in three of these Community Types. Our results are consistent with emergent neutrality, whereby ecologically specialized morphologies evolve as a consequence of neutral (stochastic or reproductive) processes within niches, leading to generalization within the frond-dominated Community Type. Our results provide an ecological signature of the first origination and subsequent loss of disparate morphologies, probably as a consequence of community restructuring in response to ecological innovation. This restructuring led to the survival of non-tiered frondose generalists over tiered specialists, even into the youngest Ediacaran assemblages. Such frondose body plans also survive beyond the Ediacaran–Cambrian transition, perhaps due to the greater resilience afforded to them by their alternative ecological strategies.

## Abstract (web summary)

The authors investigate epifaunal tiering and ecological succession within the oldest preserved animal communities, those of the Ediacaran Avalon assemblage (574–560 Ma), identifying four community types that form irrespective of succession and finding that tiering is prevalent in three community types.

## Main

Metazoans are first found widespread in the fossil record in the terminal Ediacaran, and form the Avalon assemblage (574–560 million years ago (Ma))^1–3^. Avalon communities are dominated by ‘fractally branching’ rangeomorphs and arboreomorphs^4–6^, which represent the first radiation of animal body plans^1,7,8^, alongside smaller populations of crown-group cnidaria^9^ and porifera^10,11^. However, studying the evolutionary drivers of these early animal communities is made difficult by the rarity of clear homologies between Avalon and extant organisms. An alternative approach is to directly study the ecological structure (for example, community composition, patterns of interactions and associations between taxa) of these early communities, due to their near-census-style preservation^12^; the determination of ecological structure within communities can enable the inference of underpinning ecological processes, which in turn determine evolutionary drivers^13,14^. Tiering has been suggested as an ecological mechanism structuring Avalon communities whereby species occupy different vertical spaces as a consequence of resource partitioning^15,16^, as seen in younger palaeocommunities^17–19^. Tiering has been suggested to increase throughout succession in Avalon communities, with tiering most prevalent in later-stage communities^15,20^, driving structural changes and potentially shaping the evolution of large body size and frond morphology, for example, stem evolution^16^. Yet tests of size structuring in Avalon communities have found limited prevalence of tiering—that the presence of stems decreases rather than increases community tiering, and that instead height served to increase dispersal distances—but this research only considered three surfaces^21^. More generally, Avalon communities have been demonstrated to be mostly structured by stochastic, reproductive processes^21–23^ rather than resource competition, which was relatively weak and rare^23^. Dominance of stochastic, reproductive processes suggests a stochastic mode of community succession whereby taxa accumulated to form a random composition rather than deterministic compositional transitions^24^, and so calls into question the extent to which systematic community development may have occurred in the Avalon.

**Extended Data Fig. 1:**
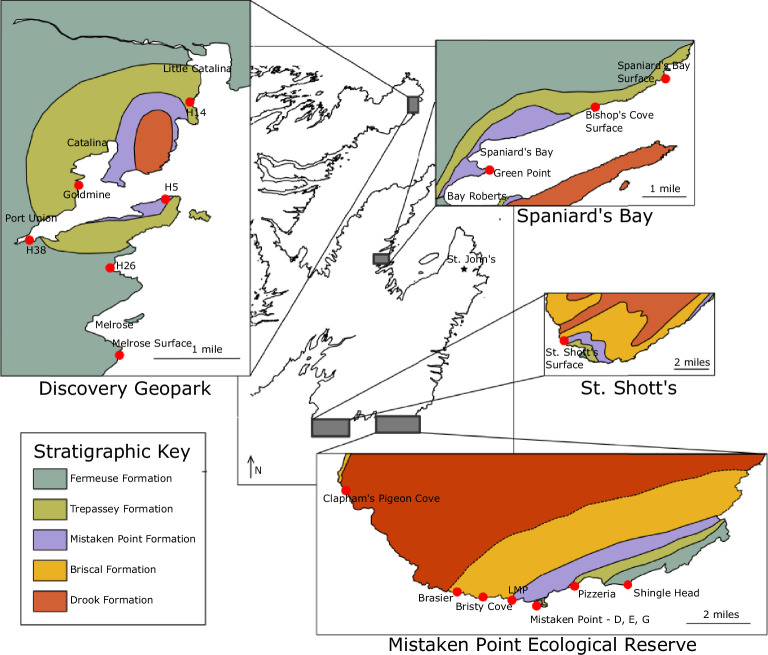
Map of fossil localities in Newfoundland, Canada. Red dots indicate location of each site, stratigraphy taken from^67^ for Mistaken Point Ecological Reserve^79^, for St. Shott’s^80^, for Spaniard’s Bay, and ^69^ for Discovery Geopark. Dashed lines indicate formation boundaries, continuous lines indicate faults.

**Extended Data Fig. 2:**
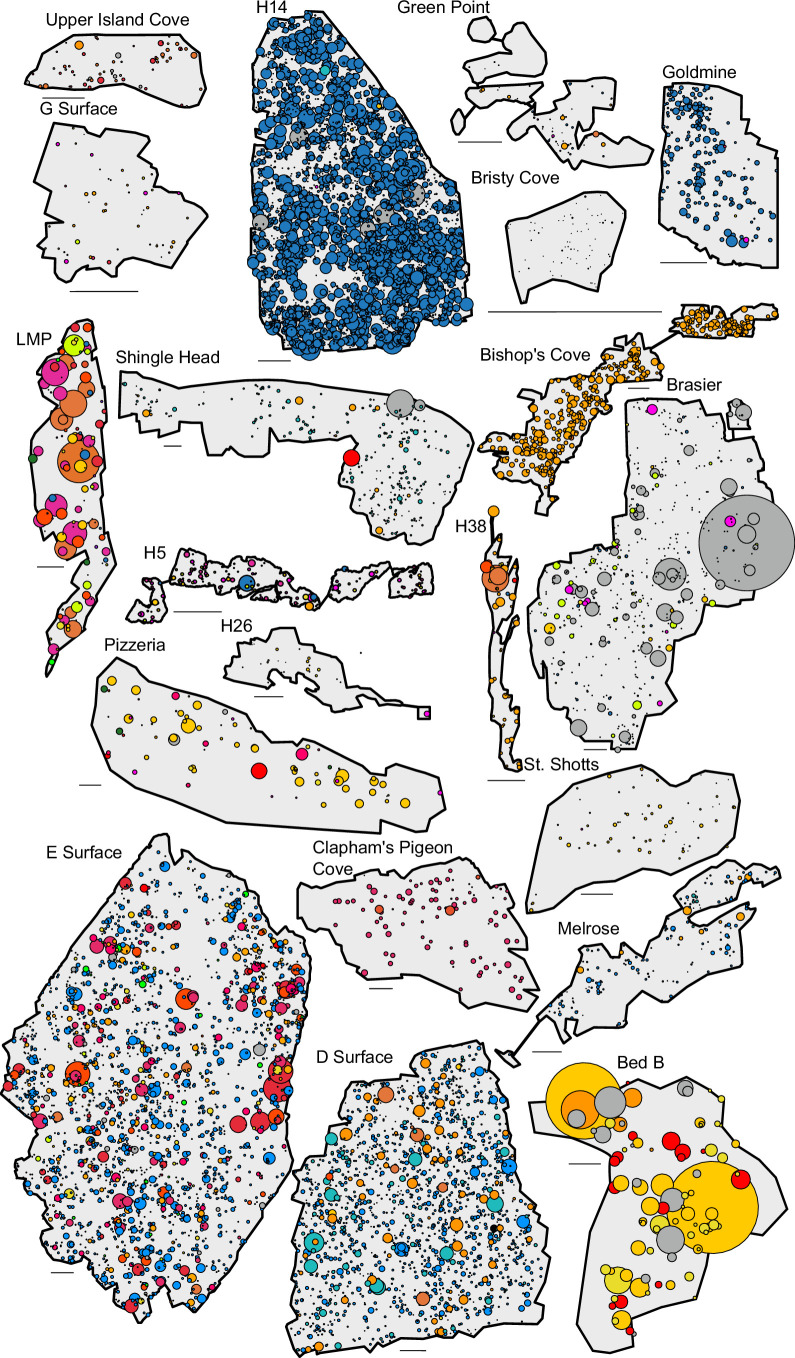
Retrodeformed maps of each fossil surface included in this study. Colour of point indicates taxon (blues = *Fractofusus* spp.; turquoise = *Pectinifrons*; greens = *Culmofrons, Frondophyllas, Plumeropriscum*; red = *Beothukis, Charniodiscus* spp.*, Thectardis*; oranges = *Bradgatia* spp.*, Vinlandia, Trepassia*; yellows = *Charnia, Primocandelabrum* spp.; purple = ostrich feather; grey = unknown/unnamed taxa) and size of point indicates frond length of specimen (frond width for *Fractofusus*). Name and scale bar for each surface given in the top and bottom left, respectively.

In this Article, we quantify the degree of succession in 18 Avalon palaeocommunities (Extended Data Fig. 1) and investigate how patterns in community composition and tiering changed throughout succession to identify the ecological processes underpinning the structure of early animal communities. We used published (five surfaces), revised (two surfaces) and new data (11 surfaces) to total 18 Avalonian communities (Extended Data Fig. 2). Data were collected using both photogrammetry and light detection and ranging (cf. ref. ^23^) to produce two-dimensional vector maps of the spatial position, disc length and width, stem and frond length and width, and identification of each specimen to species level (where possible), alongside laser line probe scans of five surfaces.

## Community composition

**Fig. 1:**
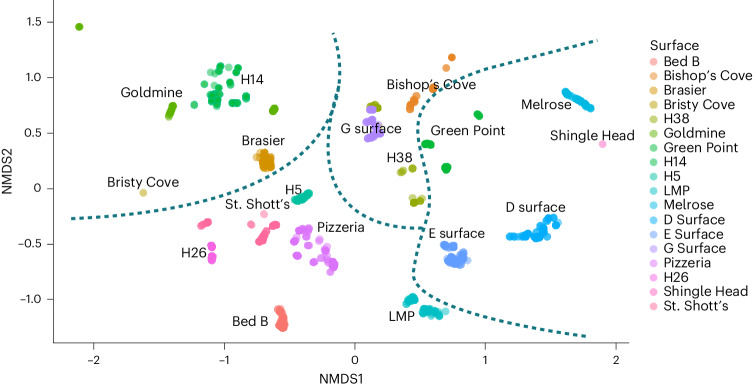
Bray–Curtis two-dimensional NMDS ordination plot of palaeocommunities (100 jackknife subsamples per community are plotted). Note that Bristy Cove (yellow/brown) and Shingle Head (pink) only have one point due to small size and tectonic deformation, respectively. Jackknife samples were aggregated by community, with overlap only between H38 and G Surface. The lines represent groupings suggested by hierarchical clustering (Fig. 2).

**Extended Data Fig. 3:**
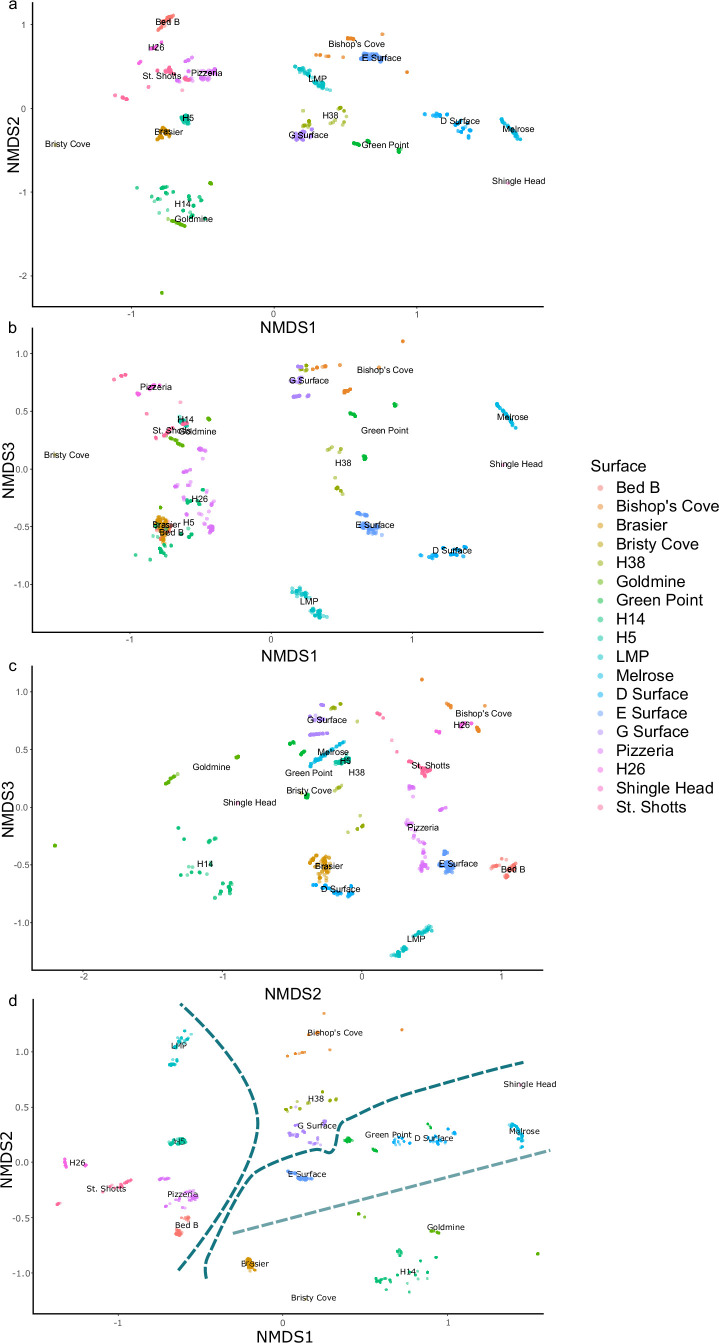
Bray-Curtis 3-dimensional non-metric multidimensional scaling ordination plots of palaeocommunities. Bray-Curtis 3-dimensional non-metric multidimensional scaling ordination plot of palaeocommunities (100 points per community shown) (stress = 0.136) with **a**) NMDS1 and NMDS2, **b**) NMDS1 and NMDS3, and **c**) NMDS2 and NMDS3 plotted. Note Bristy Cove (yellow/brown) and Shingle Head (pink) only have one point per plot. Jack-knife samples aggregate by community, with overlap only between H38 and G Surface. Lines represent groupings suggested by hierarchical clustering (Fig. 1). **d**) Bray-Curtis 2-dimensional non-metric multidimensional scaling ordination plot of palaeocommunities (first 100 points per community shown) at genus-level (stress = 0.200). Note Bristy Cove (yellow/brown) and Shingle Head (pink) only have one point. Jack-knife samples aggregate by community, with overlap only between Green Point and D Surface. Lines represent groupings suggested by hierarchical clustering, with pale line representing difference between *Fractofusus* species clusters (Fig. 2).

**Extended Data Fig. 4:**
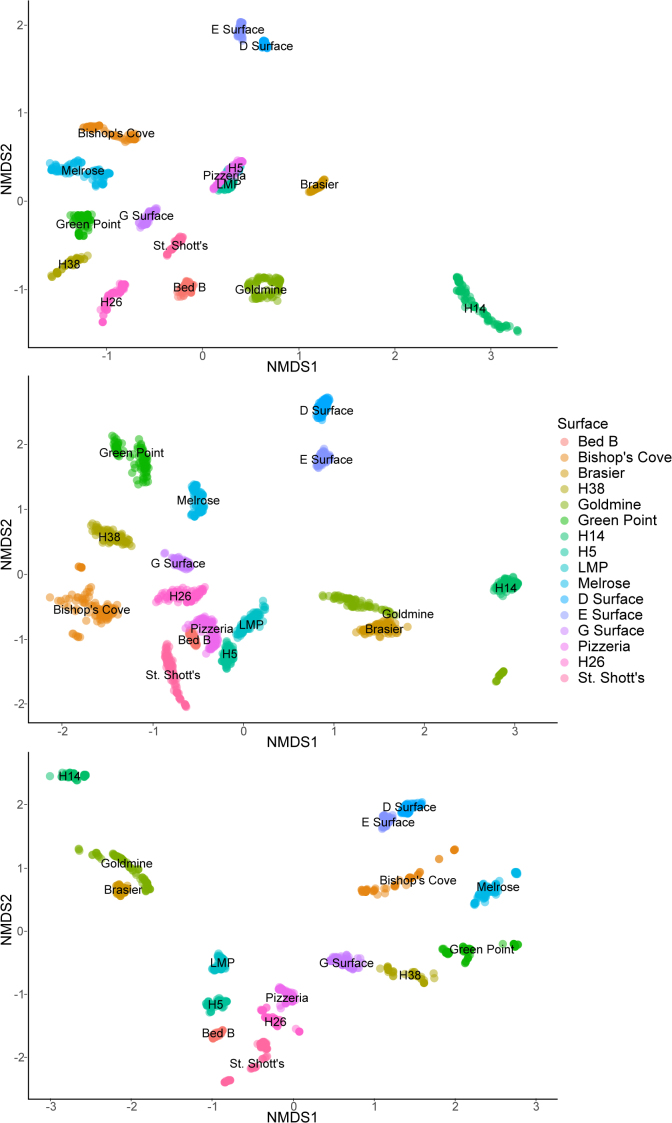
Bray-Curtis 2-dimensional non-metric multidimensional scaling ordination plots of palaeocommunities for effaced frond sensitivity analyses. Bray-Curtis 2-dimensional non-metric multidimensional scaling ordination plot of palaeocommunities (first 100 points per community shown) for effaced frond sensitivity analyses. Top: effaced frond treated as an additional taxon (stress = 0.149); middle: effaced fronds are proportionally split into taxa based on the proportions found on each surface (stress = 0.188); bottom: effaced frond identities are estimated based on gross morphology where possible, and all other taphomorphs are removed from the data (stress = 0.159).

To investigate differential successional pathways, we quantified community composition since differential pathways may be reflected in changes in community composition and/or tiering and due to influences such as differences in environment or varying source communities. To quantify variability in composition, we generated 1,000 spatial jackknife samples of 66% (ref. ^25^) for 16 out of 18 surfaces (omitting Bristy Cove and Shingle Head due to small size and tectonic deformation, respectively) by subsetting the spatial extent of each surface to 66% its total size using a random seed as a centre point 1,000 times. To visualize how communities cluster by composition, we ordinated the subsampled surface data via non-metric multidimensional scaling (NMDS) with a Bray–Curtis distance (stress of 0.196, indicating fair fit to the data), which supported the hierarchical clustering with the four groups approximately separated (Fig. 1, dashed lines). No additional structure was recovered from repeating the NMDS at three-dimensions (Extended Data Fig. 3a–c). Variation along NMDS axis 1 was associated with abundance of *Fractofusus* species (*Fractofusus misrai* positively and *Fractofusus* *andersoni* negatively) whereas NMDS axis 2 was weakly negatively associated with the relative abundance of ‘upright fronds’ (for example, *Charnia*). At the genus level, *Fractofusus* presence–absence was instead associated with NMDS1 (Extended Data Fig. 3d). We conducted sensitivity analyses of effaced fronds to reflect their high abundance on many surfaces (8 of 18 surfaces had >30% relative abundances composed of effaced fronds) and so showed increased abundance of effaced fronds associated with NMDS1, with *Fractofusus* species abundances instead associated with NMDS2 (Extended Data Fig. 4). The *Fractofusus*-dominated surfaces continued to form two clusters when subject to effaced frond sensitivity analyses, but the *Bradgatia*-dominated and ‘frond’-dominated surfaces formed one cluster (Extended Data Fig. 4), probably reflecting that these groups contribute to the effaced fronds category, unlike *Fractofusus* spp.

**Fig. 2:**
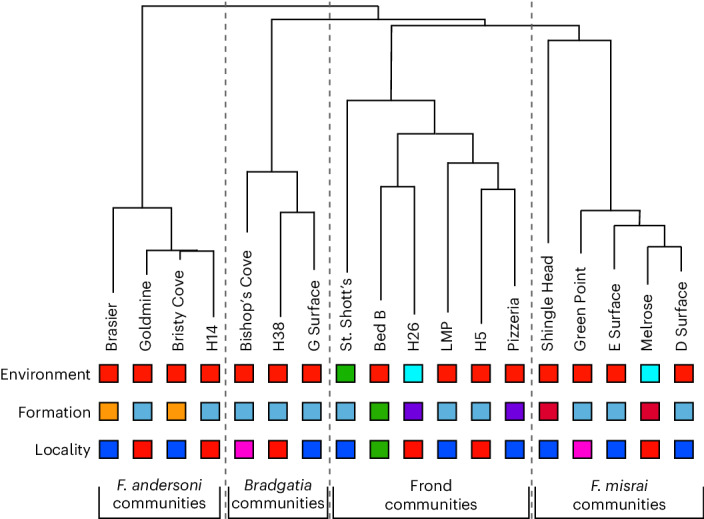
Dendrogram of hierarchical clustering of composition 18 Avalonian palaeocommunities. The dashed lines indicate qualitative clustering (shown on the NMDS by the dashed lines in Fig. 1). The colours represent groupings: environment^78^: red is margin slope, blue is outer shelf and green is unknown; formation: orange is Briscal formation, blue is Mistaken Point formation (MPER, Western Head or Bay Roberts) or Murphy’s Cove/Catalina member (Discovery Geopark), purple is Trepassey formation (MPER) or Port Union member (Discovery Geopark), red is Fermeuse formation (MPER) or Back Cove member (Discovery Geopark) and green is Blackbrook group; locality: blue is MPER and Western Head, red is Discovery Geopark, pink is Bay Roberts and green is Charnwood Forest.

**Extended Data Fig. 5:**
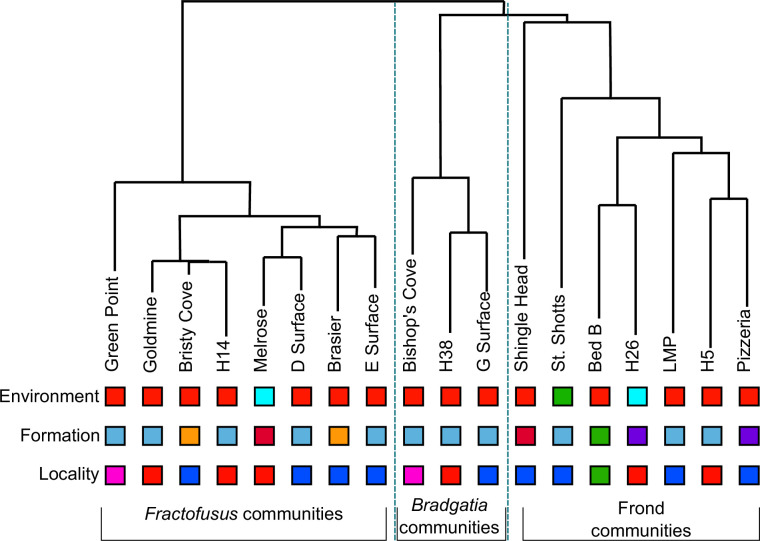
Dendrogram of hierarchical clustering of composition of Avalonian palaeocommunities at genus level. Dendrogram of hierarchical clustering of composition of 18 Avalonian palaeocommunities at genus level. Dashed lines indicate qualitative clustering. Colours represent groupings: environment: red = margin slope, blue = outer shelf, green = unknown; formation: orange = Briscal formation, blue = Mistaken Point Formation (MPER, Western Head, or Bay Roberts) or Murphy’s Cove/Catalina member (Discovery Geopark), purple = Trepassey Formation (MPER) or Port Union Member (Discovery Geopark), red = Fermeuse Formation (MPER) or Back Cove member (Discovery Geopark), green = Blackbrook group; locality: blue = MPER and Western Head, red = Discovery Geopark, pink = Bay Roberts, green = Charnwood Forest. Note the change of position of Shingle Head from species-level analyses from the *F. misrai* group to the frond group due to low sample size.

We then qualitatively investigated whether communities could be divided into types based on their composition. Using hierarchical clustering on full-surface relative abundances, we identified four distinct Community Types (with the term capitalized to distinguish it from generic ‘communities’; Fig. 2), each characterized by a different assemblage of highly abundant taxa: high relative abundance of *Bradgatia* sp. (Bishop’s Cove, H38 and G Surface; 58.3–98.6%), high relative abundance of *F. andersoni* (Brasier, Goldmine, Bristy Cove and H14; 70.1–99.6%), high relative abundance of *F. misrai* (Green Point, D Surface, E Surface and Melrose; 61.8–82.9%) and/or *Pectinifrons abyssalis* (Shingle Head; 93.7%), and high relative abundances of a range of upright frondose taxa such as *Charnia* or *Primocandelabrum* (St. Shott’s, Bed B, H5, H26, Pizzeria and Lower Mistaken Point (LMP); 92.8–100%). Community Type was not qualitatively associated with formation, geography or environmental setting at the species (Fig. 2) or genus level (Extended Data Fig. 5), with sample sizes prohibiting any formal hypothesis testing of within-group effects, for example, specific pathways within each stratigraphic unit.

## Succession analyses

**Fig. 3:**
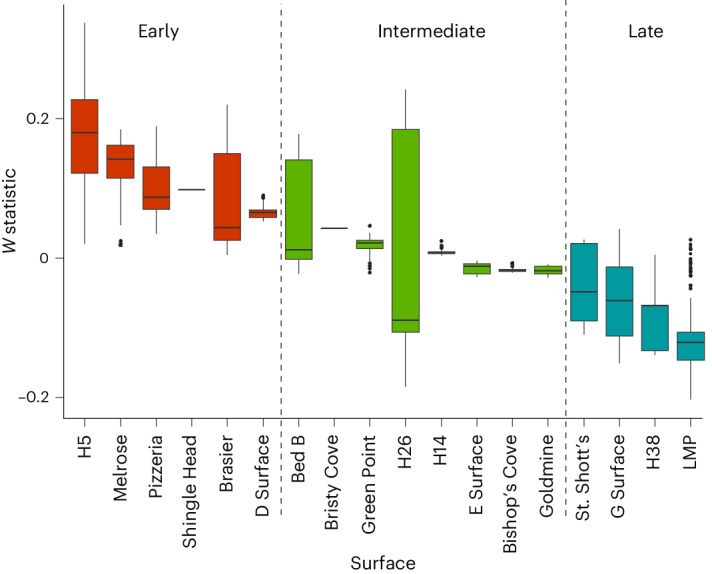
A box plot of the variation in *W* statistics across 18 Avalon palaeocommunities ordered from earlier successional stage palaeocommunities to later-stage palaeocommunities by mean *W* statistic. The bars and circles indicate medians and outliers, respectively. The boxes indicate the interquartile range (25–75th percentile) and whiskers indicate lower and upper quartiles minus or plus 1.5 times the interquartile range. The dashed lines indicate groupings of earlier (left, red), intermediate (middle, green) and later (right, blue) stage communities.

**Extended Data Fig. 6:**
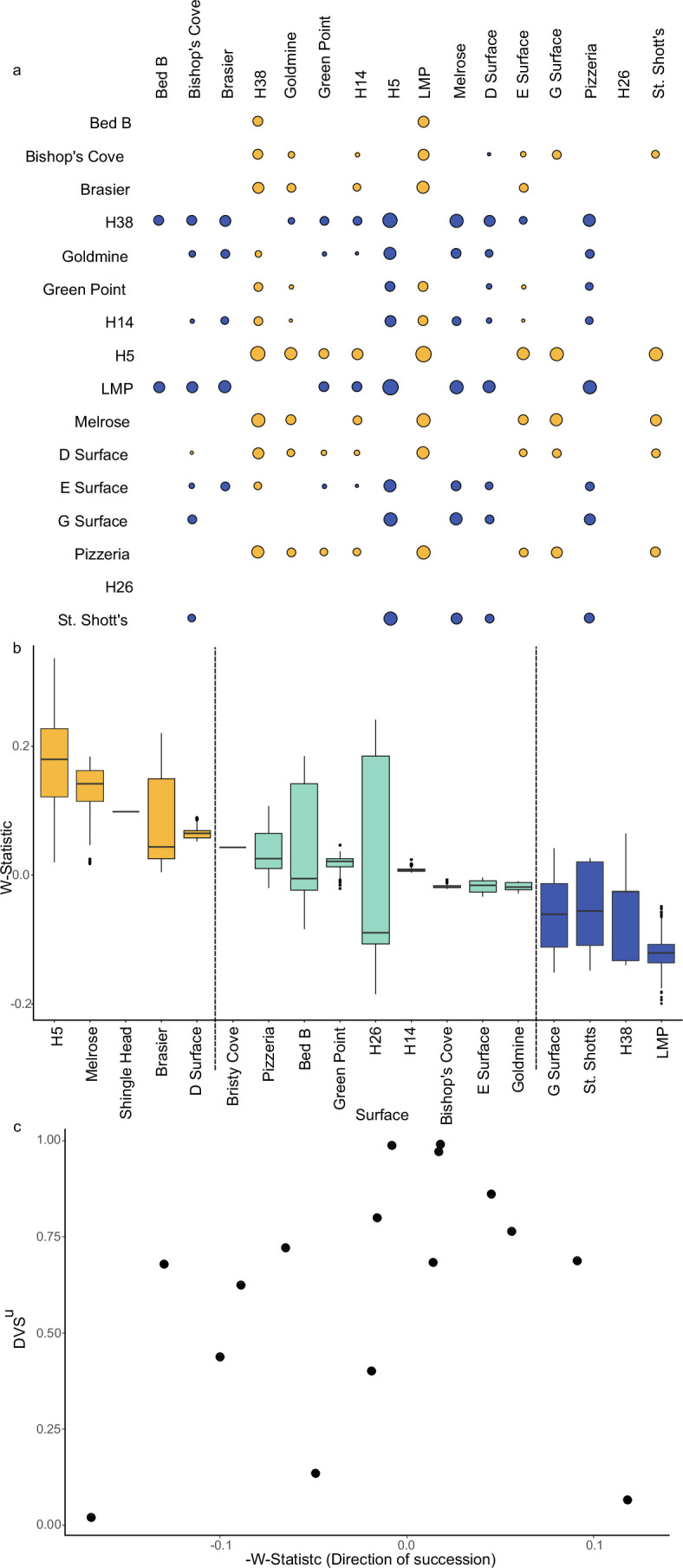
Surface comparison plots; W statistic comparisons; succession sequence at genus level; W statistic and DVSu across surfaces. **a**) Mean pairwise comparisons of the W-statistic between surface jack-knife samples. Ellipses shown are significantly different. Blue circles indicate the column is significantly later in successional stage than the row; yellow circles indicate the column is significantly earlier in successional stage than the row. Size of ellipse indicates size of mean difference. **b**) Boxplot of the variation in W-statistic across 18 Avalon palaeocommunities at genus level ordered from earlier successional stage palaeocommunities to later stage palaeocommunities by mean W-statistic. Bars and circles indicate medians and outliers, respectively. Boxes indicate the inter quartile range (25^th^–75^th^ percentile), and whiskers indicate lower and upper quartiles minus or plus 1.5 times the inter quartile range. Dashed lines indicate groupings of earlier (left), intermediate (middle), and later (right) stage communities. **c**) Non-significant relationships between W-Statistic and weighted DVS^h^ (two-sided linear regression: *p* = 0.980, adj-R^2^ = −0.056).

To quantify the degree of succession, we used abundance–biomass comparisons, which provide a measure of how recently a community was disturbed that correlates with successional stage, as communities transition from being composed of a relatively high abundance of small individuals to a relatively higher abundance of larger individuals as they develop, and the subsequent calculation of the *W* statistic^26^. The *W* statistic provides a measure between −1 and 1 of abundance–biomass trade-offs by comparing the degree of separation of abundance and biomass dominance curves, where high *W* values represent abundance dominance (earlier succession) and low represents biomass dominance (later succession)^27,28^. We calculated the *W* statistic across surface jackknife subsamples for each community to establish categorical successional stage of earlier, intermediate and later-stage communities (Extended Data Fig. 6a). We found that five surfaces always had significantly higher *W* statistics than other surfaces (H5, Pizzeria, Brasier, D Surface and Melrose; mean *W* statistic 0.250 to 0.065), so were interpreted as earlier succession. Four surfaces always had significantly lower *W* statistics than other surfaces (St. Shott’s, G Surface, H38 and LMP; mean *W* statistic −0.078 to −0.099), interpreted as later succession. Six surfaces had statistically significant differences but with higher and lower *W* statistics that other surfaces (Bed B, Green Point, H14, E Surface, Goldmine and Bishop’s Cove; mean *W* statistic 0.049 to −0.011) so were interpreted as intermediate communities. H26 was not significantly different from any other surfaces in terms of *W* statistic, but due to its mean *W* statistic being similar to intermediate-stage surfaces, we interpreted this as an intermediate-stage community. Bristy Cove (intermediate) and Shingle Head (earlier) were not subject to subsampling so were placed based on the total surface *W* statistic (Fig. 3 and Extended Data Fig. 6b). When we performed sensitivity analyses and quantified succession stage at genus level rather than species level, Bed B and G Surface changed position in the succession sequence at genus level (*x* axis, Fig. 3) but not overall stage, and Pizzeria changed to an intermediate successional stage from an early stage, probably due to its rich diversity of *Charniodiscus* species and subsequent grouping (Extended Data Fig. 6b). Sensitivity analyses of communities including effaced fronds found that the *W* statistic changed little for all but four communities (H26, H5, LMP and Melrose; mean change in *W* statistic of >0.1). Two communities did not change in degree of succession (Bed B and H14; the mean change in *W* statistic was <0.01 (Extended Data Table 1)).

We found no relationship between *W* statistic and specimen density (*N* = 16, *F*_1,16_ = 0.976, *P* = 0.338, adjusted (adj) *R*^2^ = −0.001), areal coverage (*N* = 16, *F*_1,16_ = 0.051, *P* = 0.825, adj *R*^2^ = −0.006), mean specimen height (*N* = 16, *F*_1,16_ = 1.28, *P* = 0.275, adj *R*^2^ = −0.016) and maximum specimen height (*N* = 16, *F*_1,16_ = 0.014, *P* = 0.908, adj *R*^2^ = −0.062) on any full-surface datasets using linear models.

**Fig. 4:**
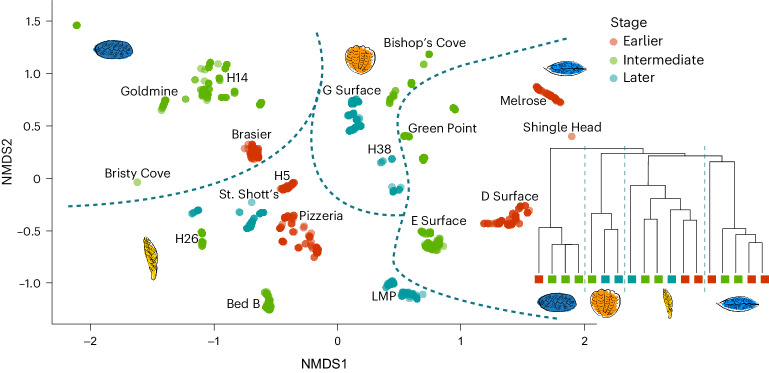
Bray–Curtis two-dimensional NMDS ordination plots of jackknife samples of palaeocommunities (100 points per community shown). Red is earlier succession, green is intermediate and blue is later succession. The lines represent groupings suggested by hierarchical clustering (Fig. 2). Dendrogram from Fig. 2 from left to right: *F. andersoni* communities, *Bradgatia* communities, Frond communities and *F. misrai* communities, with the community stages labelled.

We found no aggregation of succession stage when mapped onto an NMDS (Fig. 4) irrespective of genus level or effaced frond treatments. Instead, multiple community successional stages were present within each Community Type (Fig. 4, dashed lines) indicating no association between succession and community composition. This conclusion was confirmed by the inability of a linear discriminant analysis (LDA) to successfully predict successional stage from community composition (median correct stage prediction: 8.7%).

## Tiering

**Fig. 5:**
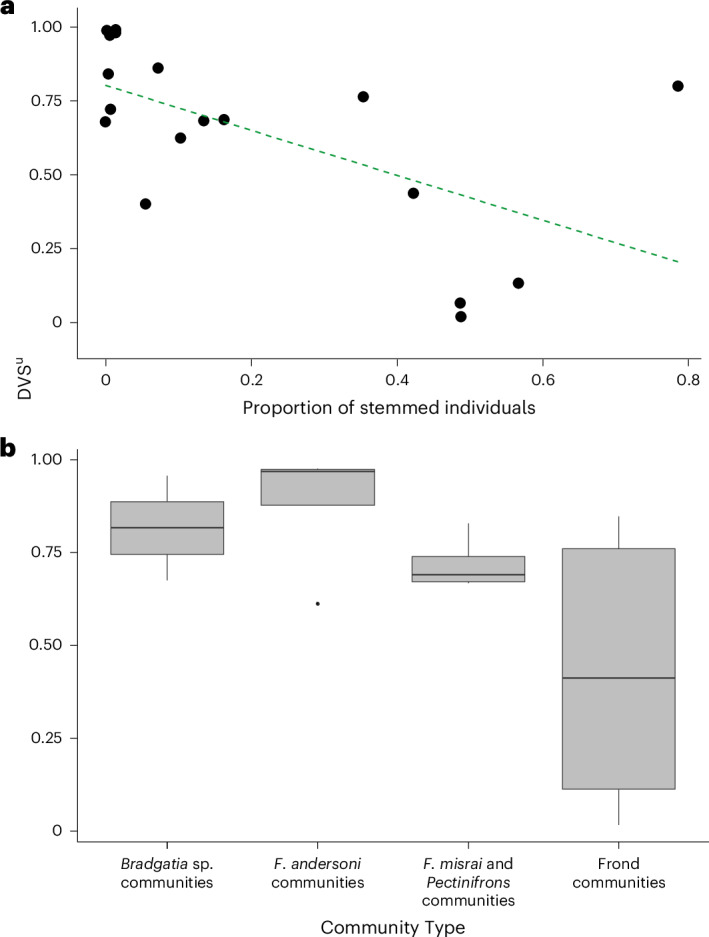
Greater DVS^u^ (*y* axis) indicates greater prevalence of tiering. **a**, Weighted DVS^u^ is significantly negatively associated with increasing proportions of stemmed taxa within a palaeocommunity. Linear regression is given by the line. **b**, DVS^u^ across Community Types. Tiering metrics are higher in communities composed primarily of *Fractofusus*, *Pectinifrons* or *Bradgatia*, than frond-dominated communities. The bars indicate median weighted DVS^u^. The boxes indicate the interquartile range (25–75th percentile) and whiskers indicate lower and upper quartiles minus or plus 1.5 times the interquartile range.

To quantify the extent of tiering in each community, we used distinct vertical stratification of the uptake (that is, feeding) zone (DVS^u^) for full surfaces as defined in ref. ^21^, but with the modification of weighting the taxon-specific DVS score by the abundance of that taxon within each community. Our weighted measure allowed us to account for variable species abundances and rarer species. We found that nine communities had high levels of tiering (>0.70), six had medium (0.40–0.70) and three low (<0.40) for DVS^u^ (Extended Data Table 1). High levels of tiering were associated with the presence of either of the two species of *Fractofusus*, which occupied a different tier to all other taxa (except *Hapsidophyllas*) and had the highest abundance in eight of 18 communities (Extended Data Table 2). To establish whether there was any association between tiering and succession between surfaces, DVS^u^ scores were compared against the *W* statistic via linear regressions, with no association found (Extended Data Fig. 6c). However, we found that the proportion of stemmed taxa was negatively correlated with weighted DVS^u^ using Spearman’s rank (*ρ*(17) = −0.670, *P* = 0.003; Fig. 5a). We also performed further analyses to enable comparisons with published work, and so tested the extent of tiering using weighted DVS^h^ and the non-weighted DVS metrics as defined in ref. ^21^ (Extended Data Table 1). We found that DVS^h^ and DVS^u^ were strongly positively correlated by Spearman’s rank (*ρ*(17) = 0.860, *P* < 0.001) and that tiering was more prevalent in weighted DVS metrics. To understand how tiering relates to different morphological traits, we split our dataset into four demonstrable ‘morphogroups’: (1) a reclining group containing *Fractofusus* and *Hapsidophyllas*, (2) *Bradgatia* sp., (3) *Pectinifrons* spp. and (4) an ‘upright frond’ group. We chose these four groups because each group represented a different gross morphology and corresponded to the dominant taxa in the Community Types identified in our hierarchical clustering (Fig. 1), and because previous tiering has only been found to be prevalent on D Surface between *Fractofusus*, *Bradgatia* and *Pectinifrons*^21^. The upright frond group contained all taxa that had a frondose morphology, including specimens with stems and stemmed individuals with multiple folia (for example, *Primocandelabrum*). We subsampled our dataset to communities that contained at least three of these morphogroups (minimum required for analyses) where abundant (*N* > 30), and investigated tiering between these morphogroups by calculating DVS scores per community. We found that DVS^u^ remained the same (mean increase of 7%), indicating robustness of tiering between morphogroups.

## Discussion

Our results show that communities in the Avalon were structured into at least four Community Types, with high levels of tiering in three of four Avalon Community Types, contra ref. ^21^. Previously performed analyses^21^ used three surfaces versus 18 here, subsampled to only contain abundant (*N* > 30) taxa, with an older taxonomic framework and did not account for species abundances. When we used the same non-weighted tiering metrics^21^ for 18 communities, we found that nine communities had high levels of tiering (>0.70), six had medium (0.40–0.70) and three low (<0.40) for at least one tiering metric (Extended Data Table 1), compared with previous results where one surface was highly tiered and two were not tiered. Thus, using the previous metrics, we find higher levels of tiering in our communities (mean DVS^u^ in ref. ^21^: 0.375 versus mean DVS^u^ in this study: 0.647). The other difference was the use of a weighted tiering metric across all taxa, which changes the relative prevalence of tiering (non-weighted DVS^u^: 11% of surfaces highly tiered, 61% not tiered; weighted DVS^u^: 50% of surfaces highly tiered, 17% not tiered). We think that this new weighted tiering metric better reflects the tiering within each community because it captures both dominant taxa (for example, *Fractofusus* in a benthic position) with high abundances, alongside absence of tiering in other, less-abundant taxa. The encapsulation of the behaviour of both enables us to detect where alternative ecological strategies may have evolved and allows for the inclusion of rare, but ecologically relevant, taxa. Therefore, the change from one (non-weighted) to nine (weighted) highly tiered communities and six medium tiered communities not only represents a refinement of how we quantify tiering, but crucially an increase of our understanding of the variation inherently present.

**Fig. 6:**
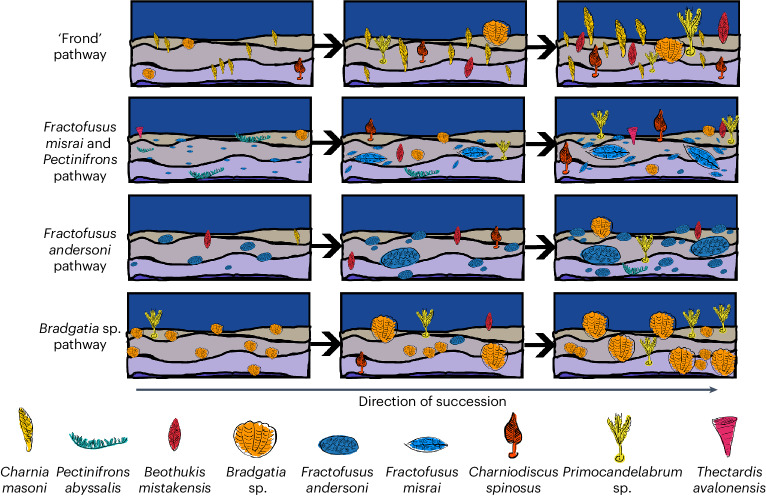
A cartoon schematic of how different Community Types underwent succession. Left to right represents earlier, intermediate and later succession communities. From top to bottom: frond communities, for example, Bed B. *F. misrai-* and *Pectinifrons*-dominated communities, for example, D Surface and E Surface. *F. andersoni*-dominated communities, for example, H14. *Bradgatia* sp.-dominated communities, for example, Bishop’s Cove.

We found that all four of the Community Types detected in this study contained multiple successional stages, indicating that succession did not necessarily lead to taxonomic turnover in the Avalon but instead that taxa persisted in relatively compositionally static communities (Fig. 6). Community Types were unlikely to be due to environmental setting, geological formation (time) or geography, as no consistent pattern comes out when these are plotted (Fig. 2). Our results provide further evidence to support aspects of previous work, which found that the abundances of *Fractofusus* and frondose taxa such as *Charnia* were key variables in the groupings of different communities^29^—we find that these taxa make up three of our four identified Community Types. Previous models of succession are also consistent with our results in the sequence of surfaces from earlier to later stages (Fig. 3 and ref. ^20^), but we find a key difference, namely no association between succession (*W* statistic) and tiering (DVS^u^) or composition, and our results are robust to sensitivity analyses.

The lack of association between succession (*W* statistic) and tiering (Extended Data Fig. 6c) indicates that Avalon communities did not get more tiered as the communities matured. We instead show that tiering was most prevalent in communities dominated by *Fractofusus, Bradgatia* or *Pectinifrons*^21^, regardless of successional stage and we found high levels of tiering in nine out of 18 surfaces. The prevalence of tiering is greater (DVS^u^ of 68.3%) on E Surface when using abundance-weighted tiering metrics compared with previous results from ref. ^21^ (DVS^u^ of 44.9%), probably because our metrics account for the dominance of taxa (namely *Fractofusus*) in the lowest tier, and also overlap of less-abundant upright fronds in higher tiers. Our results provide further evidence that stems were not associated with the prevalence of tiering in Avalon palaeocommunities (Fig. 5a) because we showed that if a community contained a larger proportion of stemmed individuals, then those individuals were more likely to occupy the same vertical space, whereas the absence of stems led to increased tiering.

### Implications of morphology-specific tiering

Morphogroup-specific tiering indicates differential vertical niche use: *Fractofusus* occupied the lowest tier alongside rare taxa such as *Hapsidophyllas*; *Bradgatia* and *Pectinifrons* both occupied the next tiers but rarely overlap vertically and a combination of ‘upright frond’ taxa form a group, irrespective of the presence of a stem, that occupy all tiers as well as further up in the water column. The high prevalence of tiered communities implies the presence of niche processes, either actively or historically, as a structuring force within Avalon communities influencing life history traits, but the lack of tiering within the ‘upright frond’ group suggests a more stochastic, neutral set of processes for frondose taxa. Lack of tiering implies that taxa with a frondose morphology are associated with neutral processes and are ecologically distinct from niche-driven, tiering taxa. The evolution of a morphology growing directly up into the water column has been associated with higher offspring dispersal ability^21^, as well ability to survive disturbances such as low-intensity, sublethal sedimentation events^30^. Therefore, adaptations to survive disturbances and reproduce to colonize post-disturbance environments could give rise to biological legacies, whereby the composition of the next generation is greatly impacted by the presence of a survivor. However, such adaptations are unlikely to generate tiering patterns within the frondose group because tiering patterns are principally generated by resource competition-associated processes^17,21^. The prevalence of reproductive lability in Avalon communities via a variety of reproductive traits^31,32^ suggests high levels of ecological redundancy whereby multiple reproductive strategies have evolved as options in case of failure or to maximize success, but are not necessarily of key importance for the current communities^31,33–35^. Therefore, the convergence of the upright frond morphology probably indicates within-morphogroup neutral processes. Ecological hypotheses such as emergent neutrality (where distinct niches containing multiple similar species emerge as a consequence of niche differentiation and within-niche neutral dynamics) may explain why multiple species exhibit similar life modes. Emergent neutrality has been found to be a highly stabilizing process, which, paired with tiering permitting taxon co-existence, could offer an explanation for the evolution of static community profiles that do not change throughout succession alongside high alpha diversity in Avalon communities^2,20,36^.

The evolution of different morphologies associated with tiering and neutral processes may have led to a group of successful organisms sharing a single life strategy upright in the water column. Taxa occupying tiers closer to the seafloor, however, have evolved alternative strategies: *Fractofusus*, for example, is almost always abundant when present^20,31^, and very few other taxa occupy its tier, indicating specialization towards a benthic position. However, as the key processes underpinning ecosystem dynamics shifted^37^, the taxa closest to the seafloor are the first to disappear from the fossil record^38^, whereas the upright frond morphology appears to survive over those closer to the seafloor throughout the Ediacaran and into the Cambrian^39–43^. The turnover of species in the lowest tiers may have been caused by the evolution of grazing^44–47^ and sediment engineering^48,49^ as species adapted to the changing seafloor. The development of such novel ecological strategies would therefore have led to a transition from the low-diversity benthic tier in the Avalon assemblage where the substrate is covered by relatively undisturbed microbial mats, to the grazing and mining of mats in the younger White Sea assemblage^45,50^ and then the partial replacement of mats and mat-associated taxa by more extensive bioturbation in the Nama assemblage and into the early Cambrian^22,37,51–55^. Thus, the lowest tier niches in the Avalon ceased to exist, leading to the extinction of the organisms specialized to these niches.

In contrast to the turnover of taxa closer to the seafloor throughout the Ediacaran, frondose morphologies survived the Ediacaran and into the early Cambrian^38,56,57^. Through generalization as a consequence of neutral processes, the frondose morphology may have had the capacity to survive the changes occurring within the niches closer to the seafloor in many different ways: through disturbance resilience, maximizing dispersal shadow, ecological redundancy and/or exaptation of other traits. Perhaps as a result of this greater resilience of the frondose morphology, this body plan continued into the Cambrian, while the body plans represented by the tiered Ediacaran taxa went extinct. Our results suggest the start of a pattern seen throughout the animal fossil record: the radiation and then extinction or survival of different morphologies in response to ecological transitions via the evolution of disparate ecological strategies.

## Methods

### Data collection

The fossils of the Avalon assemblage are found in deep-sea deposits^58,59^ preserved as near-census communities^12^, known from volcaniclastic deep-sea turbidite deposits from Newfoundland, Canada and Charnwood Forest, United Kingdom^59–61^. Minimal time averaging and limited ecological disturbance makes Avalon fossils ideal for ecological analyses (for example, ref. ^20^) as there is no evidence in these Avalon macrofossil bedding planes for mobility^62^ or sediment reworking via burrowing^63,64^. In this study, we assessed the community structure from 18 Avalonian bedding plane assemblages: Yale outcrops of the D Surface and E Surface, and the Queens outcrop of the G Surface at Mistaken Point^60,65^, Bristy Cove, LMP, Shingle Head^20^ and St. Shott’s at Western Head, Newfoundland, Canada^23^, Brasier surface^66^ and the Pizzeria surface (Fermeuse Formation, MP9 in ref. ^67^), all from the Mistaken Point Ecological Reserve (MPER), Newfoundland, Canada; Bishop’s Cove and Green Point^68^ Bay Roberts area in Newfoundland, Canada; H5, H14, H26 and H38 (ref. ^69^), the Capelin Gulch site near Melrose^70^ and Goldmine, all in the Discovery Geopark in Newfoundland, Canada; and North Quarry Bed B from Charnwood Forest, Leicestershire, United Kingdom^71,72^ (Extended Data Fig. 1). Mapped areas of fossil surfaces (Extended Data Fig. 2) were selected for large areas and consistent preservation, or for Bristy Cove and Shingle Head, inclusion in relevant previous studies^20^.

Data included in this study come from published data, revised data and new data. The data from St. Shott’s, Bed B, Bristy Cove and Spaniard’s Bay came from ref. ^23^; H14, D Surface and E Surface from ref. ^31^ and H5 from ref. ^32^ were revised to species level. New maps were made for Bishop’s Cove, Brasier, Clapham’s Pigeon Cove, Goldmine, Green Point, H26, H38, LMP, Caplin Gulch, G Surface and Pizzeria. Of these surfaces, LMP, G Surface, Clapham’s Pigeon Cove and Shingle Head have been previously mapped^20^, and were remapped due to different taxonomic definitions since their first publication, especially regarding Charniid taxa^4^. All surfaces except H38 were light detection and ranging scanned (Faro Focus^m^, mean resolution up to 0.5 mm). We collected photogrammetry data from all remapped surfaces. Additional laser scans via a laser line probe from Brasier, Pizzeria and G Surface from ref. ^23^ were used to aid with specimen identification. The total dataset comprises 18,100 specimens across 758.91 m^2^ (Extended Data Table 2).

### Data processing

Three-dimensional models of surfaces were used to create orthomosaics of the surfaces in Agisoft Metashape v1.7.5. From these orthomosaics, two-dimensional vector maps of specimens were made in Inkscape v1.3. Vector maps contained information on the spatial position, disc length and width, stem and frond length and width, and species-level identification of each specimen (Extended Data Table 3). In instances where species-level identification was not possible, specimens were binned into genus, then morpho-categories, either using identifiable features of undescribed taxa (for example, branching characters) or into higher taxonomic or taphomorphic groupings such as ‘Rangeomorph’ or ‘Ivesheadiomorph’. Data from vector maps were extracted using a custom script (https://github.com/nis38/NPS_dex) in R 4.2.2 (ref. ^73^).

### Analyses

We performed all analyses in R 4.2.2 (ref. ^73^). All surfaces were retrodeformed by restoring elongated holdfast discs >10 mm in diameter to circles^59^. There were insufficient holdfast discs to retrodeform Clapham’s Pigeon Cove^20^.

### Clustering and NMDS analyses

To quantify overall differences in community structure, we performed a hierarchical clustering analysis on the relative abundance of communities^74^. Clustering was qualitatively compared against environmental setting (where possible; data from ref. ^39^), formation (as a proxy for age) and locality (as a proxy for geography).

To conduct statistical tests and comparisons of variance between communities, we generated 1,000 spatial jackknife samples of 66% of each surface^25^ (excepting Shingle Head due to tectonic deformation, and Bristy Cove due to size). Jackknife samples were derived by subsetting each complete surface dataset to 66% of its spatial extent. The centre points of each jackknife sample were randomly seeded (that started at a random position) on the surface and an expanding box was then used until 66% of the surface was captured. If the expanding box encountered a surface edge, the box continued to expand in all other directions until 66% of the spatial area of each surface was sampled. Each jackknife sample was generated independently, and thus could overlap with other samples. Count abundance data for each jackknife sample were then ordinated via NMDS with a Bray–Curtis distance, repeated with two- and three-dimensions. No subsets or taxa were eliminated from the subsampling. NMDS describe community compositional differences, which can be compared with measures of processes such as tiering or succession. Clustering and NMDS were conducted at species and genus level.

**Extended Data Fig. 7:**
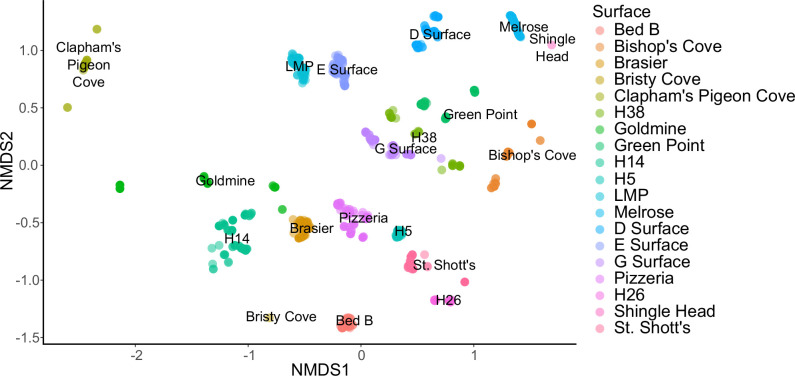
Bray-Curtis 2-dimensional non-metric multidimensional scaling ordination plot of palaeocommunities including outlier surfaces. Bray-Curtis 2-dimensional non-metric multidimensional scaling ordination plot of palaeocommunities (first 100 points per community shown) including Clapham’s Pigeon Cove, which occupied variation along NMDS1 (stress = 0.181).

To investigate the impact of effaced fronds (namely frondose specimens that could not be identified to genus level) on our analyses, we created four data treatments: (1) a ‘standard’ treatment containing no taphomorphs (all analyses were run on this treatment), (2) a treatment containing all effaced fronds as an additional ‘taxon’, (3) a treatment where effaced fronds were randomly relabelled as taxa identified in the community keeping to the proportions each taxon (that is, in a community where *Charnia* made up 50% of the composition, 50% of effaced fronds would be relabelled *Charnia*) and (4) a treatment where effaced fronds were identified to a ‘best guess’ of their identity where possible and the remaining effaced fronds removed. NMDS was conducted with all effaced frond treatments. Analyses were repeated without outliers since these can influence variability within ordination analyses (Extended Data Fig. 7).

### Quantifying degree of succession

To quantify the degree of succession, we used abundance–biomass comparisons^27^ providing a measure of how recently a community was disturbed. Abundance–biomass comparisons correlate with successional stage as communities transition from being composed of a relatively high abundance of small individuals (with an uneven abundance distribution but an even biomass distribution), to a community with relatively higher abundance of larger individuals as they develop (uneven biomass distribution and even abundance distribution) and subsequent calculation of the *W* statistic^26,28^. The *W* statistic provides a measure between −1 and 1 of abundance–biomass trade-offs by comparing abundance and biomass dominance curves, where a high *W* statistic represents abundance dominance (earlier succession) and a low *W* statistic represents biomass dominance (later succession). We used areal coverage as a proxy for biomass because of the established correlation between size and biomass in deep-sea invertebrates^75^. For each of the 1,000 jackknife samples for each surface, the *W* statistic of each sample was compared between surfaces via a matrix of distances (Extended Data Fig. 6a). Confidence intervals (alpha of 0.05) for distances of the *W* statistics for each surface were then calculated. If the confidence intervals for the distance of the *W* statistic across all subsamples crossed 0, then the difference in successional stage between any two surfaces was not considered statistically significantly different. Abundance–biomass comparisons were conducted at species and genus level.

The mean *W* statistic was compared against specimen density, total specimen areal coverage, mean specimen height and maximum specimen height for each palaeocommunity using linear regression. Regressions were conducted at the species and genus level.

To add support to any succession-driven structure detected within NMDS, an LDA was modelled with successional stage as the predictor variable and the relative abundance of each taxon as the explanatory variables. LDA detects linear combinations of features (here, species relative abundances) to establish whether these combinations underpin differences between groups (here, successional stage), differing from NMDS, which uses a non-linear ordination algorithm to cluster samples (here, jackknife samples of each surface) by similar features (here, Bray–Curtis-transformed abundances). While NMDS clusters data by common species into groupings, LDA is a supervised approach whereby groups are sought via linear combinations of species, which we use to emphasise the species that distinguish between the groupings^76^. The fit of the LDA was assessed using a split testing–training dataset where the testing data represented the jackknife samples of three randomly selected subsampled surfaces. LDA was conducted at species and genus level and with all effaced frond treatments. Successional stage was visually compared against NMDS and clustering analyses.

### Quantifying tiering

We quantified the extent of tiering using DVS metrics defined as per ref. ^21^ as the percentage of specimens within each taxon population where the ‘uptake zone’ (branching part) of the organism do not occupy the same vertical space in the water column (DVS_uptake_ in ref. ^21^). Two DVS metrics were calculated per taxon and then the mean was taken across each community: (1) DVS^h^ (the proportion of specimens from each taxon which are not matched in height by any other taxa) and (2) DVS^u^ (the proportion of ‘uptake zone’ (for example, branching) of each taxon that does not overlap with the ‘uptake zone’ of any other taxa). Height for reclining taxa was taken as one-third of the width cf. ^21^. We refined these DVS metrics by producing a weighted DVS metric by weighting mean taxon-specific DVS scores using the abundance of that taxon. This weighted metric meant that we were able to include rare taxa instead of limiting our data to common species, thus better representing the communities. To establish whether there was any association between tiering and succession between surfaces, DVS and weighted DVS scores were compared against the *W* statistic via linear regressions. DVS analyses were conducted at the species and genus level.

To understand the nature of tiering within communities, we split our dataset into four demonstrable ‘morphogroups’: (1) a reclining group containing *Fractofusus* and *Hapsidophyllas*, (2) *Bradgatia* sp., (3) *Pectinifrons* spp. and (4) an ‘upright frond’ group. We chose these four groups because each group represented a different gross morphology, and previous tiering has been found to be prevalent on D Surface between *Fractofusus*, *Bradgatia* and *Pectinifrons*^21^. The upright frond group contained all taxa that had a frondose morphology including specimens with stems and stemmed individuals with multiple folia (for example, *Primocandelabrum*). We then subsampled our dataset to communities that contained at least three (minimum required for analyses) of these morphogroups that were abundant (*N* > 30) and investigated tiering between these groups by calculating weighted DVS scores per community.

### Reporting summary

Further information on research design is available in the Nature Portfolio Reporting Summary linked to this article.

### Supplementary information

Reporting Summary

## Data Availability

Access to the fossil localities is by scientific research permit only. The data required to reproduce the results of this study are available at: https://figshare.com/s/922c126c4282f5390dec (ref. ^77^).

## Extended data

**Extended Data Table 1:** Standard and weighted DVS^h^ and DVS^u^ statistics for each surface and effaced frond treatment W statistics

DVS Statistics						Mean W Statistics		
Surface	DVS^h^	DVS^u^	Weighted DVS^h^	Weighted DVS^u^	No effaced fronds	Effaced fronds as additional taxon	Effaced fronds proportionally relabelled	‘Best guess’ applied to effaced fronds where possible
Bed B	0.065	0.168	0.061	0.134	0.049	0.033	0.048	0.042
Bishop’s Cove	0.246	0.246	0.972	0.972	−0.017	−0.074	−0.135	−0.026
Brasier	0.177	0.282	0.714	0.624	0.089	0.057	−0.015	0.045
Bristy Cove	1	0.826	1	0.981	–	–	–	–
Goldmine	0.245	0.749	0.962	0.991	−0.018	−0.096	−0.157	−0.04
Green Point	0.458	0.225	0.636	0.401	0.019	−0.003	−0.076	0
H14	0.139	0.233	0.993	0.988	0.008	0.004	0.001	0.007
H26	0.573	0.670	0.643	0.800	0.016	−0.239	−0.066	0.067
H38	0.324	0.450	0.612	0.687	−0.091	−0.118	−0.195	−0.097
H5	0.058	0.022	0.111	0.020	0.169	0.429	0.274	0.178
LMP	0.146	0.083	0.091	0.066	−0.118	0.186	0.082	−0.023
Melrose	0.331	0.283	0.655	0.679	0.13	0.008	0.007	0.055
D Surface	0.104	0.260	0.686	0.722	0.065	0.019	0.029	0.022
E Surface	0.049	0.102	0.528	0.683	−0.014	−0.013	−0.068	−0.016
G Surface	0.485	0.636	0.500	0.764	−0.056	0.094	−0.002	−0.034
Pizzeria	0.226	0.194	0.282	0.438	0.1	0.137	0.079	0.026
Shingle Head	0.638	0.508	0.919	0.841	–	–	–	–
St. Shott’s	0.217	0.425	0.768	0.861	−0.045	−0.036	−0.031	−0.058

W-statistics taken across 1,000 spatial jack-knife subsamples.

**Extended Data Table 2:** Summary data for surfaces included in this study

Surface	Area mapped (m^2^)	Total number of specimens	Density (spec/m^2^)	Most abundant taxon
Bed B	115.16	757	6.57	*Primocandelabrum sp*.
Bishop’s Cove	38.66	531	13.74	*Bradgatia sp*.
Brasier (BR5)	40.88	1032	25.24	*Fractofusus andersoni*
Bristy Cove	0.81	117	144.44	*Fractofusus andersoni*
Clapham’s Pigeon Cove	33.53	422	12.58	*Thectardis avalonensis*
Goldmine	14.05	320	22.78	*Fractofusus andersoni*
Green Point	8.24	131	15.90	*Fractofusus misrai*
H14 (Johnson Discovery Surface)	69.06	4070	58.93	*Fractofusus andersoni*
H5	4.11	539	131.14	*Charnia masoni*
H26	6.57	100	15.22	*Primocandelabrum sp*.
H38	7.94	98	12.35	*Bradgatia sp*.
LMP	21.30	566	26.57	*Beothukis sp*.
Melrose (Capelin Gulch)	11.38	222	19.51	*Fractofusus misrai*
D Surface	70.89	2289	32.29	*Fractofusus misrai*
E Surface	65.42	5085	77.73	*Fractofusus misrai*
G Surface	7.54	170	22.55	*Bradgatia sp*.
Pizzeria (MP9)	31.69	764	24.11	*Charnia masoni*
Shingle Head	144.35	411	2.85	*Pectinifrons abyssalis*
St. Shott’s	50.93	470	9.23	*Charnia masoni*
Spaniards Bay	16.40	64	3.90	*Trepassia wardae*

Most abundant taxon is the most abundant fossil identifiable to genus/species level and does not include taphomorphs.

**Extended Data Table 3:** Taxonomic identification details

Specimen ID	Description
*Arborea arborea*	cf. Fig. 1C in^81^
*Auroralumina attenboroughii*	cf.^9^
*Avalofractus abaculus*	cf.^82^
*Beothukis mistakensis*	cf.^83^
*Bradgatia linfordensis*	cf.^71^
*Bradgatia* sp.	Similar to *Bradgatia linfordensis*, generally smaller with fewer branches, found in Newfoundland
‘Brush’	Unident. taxon on Bed B
*Charniodiscus arboreus*	cf.^84^
*Charniodiodiscus procerus*	cf.^84^
*Charniodiscus spinosus*	cf.^84^
*Charniodiscus* sp.	Wastebasket taxon of unident. Arboreomorphs
*Charnia masoni*	cf.^72^
*Culmofrons plumosa*	cf.^16^
*Fractofusus andersoni*	cf.^85^
*Fractofusus misrai*	cf.^85^
*Frondophyllas grandis*	cf.^86^
*Hadryniscala avlonica*	cf.^69^
*Hapsidophyllas flexibilis*	cf.^86^
*Hyllaecululus fordi*	cf.^81^
‘Ostrich feather’	Undescribed small frond cf.^20^ and Fig 4E in^35^
*Parviscopa bonavistensis*	cf.^69^
*Pectinifrons abyssalis*	cf.^87^
*Plumeropriscum hofmanni*	cf.^88^
*Primocandelabrum aethelflaedia*	cf.^89^
*Primocandelabrum boyntoni*	cf.^89^
*Primocandelabrum* sp.	cf.^69^
*Thecthardis avalonensis*	cf.^90^
*Trepassia wardae*	cf.^91^
*Vinlandia antecedens*	cf.^4^
‘Taxon A’	cf. Fig 5A in^81^
‘Taxon B’	Unident. rangeomorph on Brasier surface^66^
‘Taxon C’	cf. Fig. 2B in^35^
‘Taxon D’	cf. Fig 5G in^2^
‘Taxon E’	Unident. rangeomorph, similar to ‘Taxon C’
‘Taxon F’	Unident. rangeomorph on E Surface
‘Taxon G’	Unident. rangeomorph on E Surface
‘Taxon H’	Unident. *Pectinifrons*-like specimen on Pizzeria
‘Taxon I’	Unident. unifoliate rangeomorph on Shingle Head

## Code availability

The code required to reproduce the results of this study has been uploaded to: https://github.com/nis38/Morphology_Comm_Dyn and https://github.com/nis38/NPS_dex.
