# Author Correction: Morphology shapes community dynamics in early animal ecosystems

**DOI:** 10.1038/s41559-024-02521-6

**Published:** 2024-07-30

**Authors:** Nile P. Stephenson, Katie M. Delahooke, Nicole Barnes, Benjamin W. T. Rideout, Charlotte G. Kenchington, Andrea Manica, Emily G. Mitchell

**Affiliations:** 1 Department of Zoology, University of Cambridge, Cambridge, UK; 2 University Museum of Zoology, University of Cambridge, Cambridge, UK; 3 Department of Earth Sciences, University of Cambridge, Cambridge, UK; 4 Independent Researchers, https://www.nature.com/natecolevol/

**Keywords:** Community ecology, Palaeontology, Palaeoecology

Correction to: *Nature Ecology & Evolution* 10.1038/s41559-024-02422-8, published online 12 June 2024.

In the version of the article initially published, an error in the code resulted in an incorrect calculation of tiering. This has now been corrected, and changes have been made to the main text, Discussion, Fig. 5, Extended Data Table 1 and Extended Data Table 6 (see the accompanying Supplementary information for a detailed description of changes). All other analyses and results are unaffected by this mistake.

## Supplementary information

Detailed changes and original figures

