# Supplementary material for: Morphology shapes community dynamics in early animal ecosystems
Source: Nat Ecol Evol. 2024 Jun 12;8(7):1238–47. doi: 10.1038/s41559-024-02422-8 (PMC11239517; doi:10.1038/s41559-024-02422-8)
Supplement: Supplementary file 1 — Reporting Summary [file 41559_2024_2422_MOESM1_ESM.pdf]

nature portfolio

Corresponding author(s): Nile Stephenson

Last updated by author(s): 2024/04/17

Reporting Summary

Nature Portfolio wishes to improve the reproducibility of the work that we publish. This form provides structure for consistency and transparency in reporting. For further information on Nature Portfolio policies, see our Editorial Policies and the Editorial Policy Checklist.

Statistics

For all statistical analyses, confirm that the following items are present in the figure legend, table legend, main text, or Methods section.

n/a	Confirmed	
☐	☒	The exact sample size (*n*) for each experimental group/condition, given as a discrete number and unit of measurement
☐	☒	A statement on whether measurements were taken from distinct samples or whether the same sample was measured repeatedly
☐	☒	The statistical test(s) used AND whether they are one- or two-sided<br>*Only common tests should be described solely by name; describe more complex techniques in the Methods section.*
☐	☒	A description of all covariates tested
☒	☐	A description of any assumptions or corrections, such as tests of normality and adjustment for multiple comparisons
☐	☒	A full description of the statistical parameters including central tendency (e.g. means) or other basic estimates (e.g. regression coefficient) AND variation (e.g. standard deviation) or associated estimates of uncertainty (e.g. confidence intervals)
☐	☒	For null hypothesis testing, the test statistic (e.g. *F*, *t*, *r*) with confidence intervals, effect sizes, degrees of freedom and *P* value noted<br>*Give P values as exact values whenever suitable.*
☒	☐	For Bayesian analysis, information on the choice of priors and Markov chain Monte Carlo settings
☒	☐	For hierarchical and complex designs, identification of the appropriate level for tests and full reporting of outcomes
☒	☐	Estimates of effect sizes (e.g. Cohen's *d*, Pearson's *r*), indicating how they were calculated

Our web collection on statistics for biologists contains articles on many of the points above.

Software and code

Policy information about availability of computer code

Data collection	No software was used during data collection for this study.
Data analysis	R v4.2.1, Agisoft Metashape V2.0.2, Inkscape v1.1, GeoMagic Wrap (2015, 2017), Faro SCENE

For manuscripts utilizing custom algorithms or software that are central to the research but not yet described in published literature, software must be made available to editors and reviewers. We strongly encourage code deposition in a community repository (e.g. GitHub). See the Nature Portfolio guidelines for submitting code & software for further information.

Data

Policy information about availability of data

All manuscripts must include a data availability statement. This statement should provide the following information, where applicable:

- Accession codes, unique identifiers, or web links for publicly available datasets
- A description of any restrictions on data availability
- For clinical datasets or third party data, please ensure that the statement adheres to our policy

Data for this study is available via the figshare link (https://figshare.com/s/922c126c4282f5390dec) provided in the manuscript. We provided data for the first 100 jack knife subsamples of all surfaces in this study instead of all 1000 subsamples due to constraints on the size of data storage, but all data can be provided upon request. Due to geoconservation concerns, data from Bed B is not publicly available, with the full dataset available to researchers upon request from the corresponding author. No data collected for this study uses accession codes or is publicly available.

Research involving human participants, their data, or biological material

Policy information about studies with human participants or human data. See also policy information about sex, gender (identity/presentation), and sexual orientation and race, ethnicity and racism.

Reporting on sex and gender	*Use the terms sex (biological attribute) and gender (shaped by social and cultural circumstances) carefully in order to avoid confusing both terms. Indicate if findings apply to only one sex or gender; describe whether sex and gender were considered in study design; whether sex and/or gender was determined based on self-reporting or assigned and methods used. Provide in the source data disaggregated sex and gender data, where this information has been collected, and if consent has been obtained for sharing of individual-level data; provide overall numbers in this Reporting Summary. Please state if this information has not been collected. Report sex- and gender-based analyses where performed, justify reasons for lack of sex- and gender-based analysis.*
Reporting on race, ethnicity, or other socially relevant groupings	*Please specify the socially constructed or socially relevant categorization variable(s) used in your manuscript and explain why they were used. Please note that such variables should not be used as proxies for other socially constructed/relevant variables (for example, race or ethnicity should not be used as a proxy for socioeconomic status). Provide clear definitions of the relevant terms used, how they were provided (by the participants/respondents, the researchers, or third parties), and the method(s) used to classify people into the different categories (e.g. self-report, census or administrative data, social media data, etc.) Please provide details about how you controlled for confounding variables in your analyses.*
Population characteristics	*Describe the covariate-relevant population characteristics of the human research participants (e.g. age, genotypic information, past and current diagnosis and treatment categories). If you filled out the behavioural & social sciences study design questions and have nothing to add here, write "See above."*
Recruitment	*Describe how participants were recruited. Outline any potential self-selection bias or other biases that may be present and how these are likely to impact results.*
Ethics oversight	*Identify the organization(s) that approved the study protocol.*

Note that full information on the approval of the study protocol must also be provided in the manuscript.

Field-specific reporting

Please select the one below that is the best fit for your research. If you are not sure, read the appropriate sections before making your selection.

☐ Life sciences ☐ Behavioural & social sciences ☒ Ecological, evolutionary & environmental sciences

For a reference copy of the document with all sections, see nature.com/documents/nr-reporting-summary-flat.pdf

Ecological, evolutionary & environmental sciences study design

All studies must disclose on these points even when the disclosure is negative.

Study description	We studied the factors underpinning community structure by investigating succession and tiering processes in Avalon palaeocommunities. We collected census data via photogrammetry and laser scanning fossiliferous bedding planes. This study uses data from 18 such bedding planes, featuring 18100 specimens across 758.91 square metres of rock surface. From photogrammetry, we extracted size and abundance data from these palaeocommunities, and then used mathematical models to investigate patterns in succession and tiering in these data to see if this explained community composition.
Research sample	Previously published and newly collected data were used in this study. Collected data included photogrammetry of Ediacaran fossiliferous bedding planes in Newfoundland, Canada, which did not involve removal of any material, and LiDAR scans of fossils beds. Previously collected data also included photogrammetry of bedding plans from Newfoundland, Canada, as well as LiDAR and laser line probe scans of surfaces and Reflectance Transformation Imaging of casts from one surface in Leicestershire, UK.
Sampling strategy	Sampling involved capture of full census communities. We specifically selected surfaces which has limited geological biases (e.g. differential erosion) and were relatively large, or were involved in relevant previous studies (noted in manuscript). In order to evaluate variability in the data, we spatially jack-knifed each bedding plane dataset 1,000 times.
Data collection	Data were collected for this study using photogrammetry and LiDAR of 11 bedding plane surfaces in Newfoundland, Canada. Photogrammetry data were collected by taking still photographhs in a boustrophedonic grid over the target rock surface in order to generate a 3D model and 2D map of the subject. Photogrammetry data for new data in the study were collected by Dr Emily Mitchell, Dr Charlotte Kenchington, Katie Delahooke, and Nile Stephenson. Processing of photogrammetry data occurred in Agisoft Metashape, LiDAR scans of data used in the study were collected using Faro Focus m by Dr Emily Mitchell, Katie Delahooke, and Nile Stephenson. Post-processing of data in GeoMagic and Agisoft Metashape was conducted by Dr Emily Mitchell, Dr Charlotte Kenchington, Katie Delahooke, Nicole Barnes, and Nile Stephenson.
Timing and spatial scale	We selected the largest fossil surfaces to study to maximise sample size of census communities. Fossil surfaces with significant erosional biases were not considered.

Data exclusions	We excluded two bedding planes from our analyses; Upper Island Cove was excluded because of recent publications questioning the sedimentology of the surface, which might indicate that these data are no necessarily appropriate for ecological analyses. Clapham's Pigeon Cove was excluded from analyses in the main text because it has a unique assemblage and is by far the oldest community, and so was classed as an outlier. We do, however, include results of analyses including Clapham's Pigeon Cove in the SI.
Reproducibility	Reproducible code and data have been provided on github and figshare, respectively. Data from Bed B cannot be shared in the interest of protecting geological material.
Randomization	No randomisation occurred in this study as we were intentionally working with whole census data.
Blinding	Blinding was not relevant to this study because we were working with whole census data.

Did the study involve field work? ☒ Yes ☐ No

Field work, collection and transport

Field conditions	Fieldwork was conducted in dry, usually sunny conditions in Newfoundland, Canada in June, July, August, and September, where weather conditions are fair (12 - 22 degrees Celcius). Fieldwork cannot be conducted in rain or fog because this does not permit photogrammetry data collection.
Location	Newfoundland, Canada
Access & import/export	No geological material was removed for this study. We collected photogrammetry data using still photography from fieldsites. Access to fieldsites was granted and conducted with permission in Mistaken Point Ecological Reserve (MPER_2016-19, and MPER_2021-23) and under permit from the Government of Newfoundland and Labrador for Paleontological Investigation (P16.04, P17.01; P18.01, P19.01, P21.01, and P23.01) in Bonavista Peninsula, around Port Union and Little Catalina, Spaniard's Bay, and St. Shotts.
Disturbance	None; no material was removed in this study. We followed regulations put in place by governing bodies to protect fossil surfaces (appropriate footware, rubber endings on tripod legs).

Reporting for specific materials, systems and methods

We require information from authors about some types of materials, experimental systems and methods used in many studies. Here, indicate whether each material, system or method listed is relevant to your study. If you are not sure if a list item applies to your research, read the appropriate section before selecting a response.

Materials & experimental systems

n/a	Involved in the study	
☒	☐	Antibodies
☒	☐	Eukaryotic cell lines
☐	☒	Palaeontology and archaeology
☒	☐	Animals and other organisms
☒	☐	Clinical data
☒	☐	Dual use research of concern
☒	☐	Plants

Methods

n/a	Involved in the study	
☒	☐	ChIP-seq
☒	☐	Flow cytometry
☒	☐	MRI-based neuroimaging

Palaeontology and Archaeology

Specimen provenance	No specimens were removed or used in this study, but permits were required to work on the fossil surfaces in Newfoundland, Canada.
Specimen deposition	No specimens were used in this study.
Dating methods	No dates were ascertained in this study.

☐ Tick this box to confirm that the raw and calibrated dates are available in the paper or in Supplementary Information.

Ethics oversight	No ethical approval or guidance was required in this study because we were not removing fossiliferous material.

Note that full information on the approval of the study protocol must also be provided in the manuscript.

Plants

Seed stocks	*Report on the source of all seed stocks or other plant material used. If applicable, state the seed stock centre and catalogue number. If plant specimens were collected from the field, describe the collection location, date and sampling procedures.*
Novel plant genotypes	*Describe the methods by which all novel plant genotypes were produced. This includes those generated by transgenic approaches, gene editing, chemical/radiation-based mutagenesis and hybridization. For transgenic lines, describe the transformation method, the number of independent lines analyzed and the generation upon which experiments were performed. For gene-edited lines, describe the editor used, the endogenous sequence targeted for editing, the targeting guide RNA sequence (if applicable) and how the editor was applied.*
Authentication	*Describe any authentication procedures for each seed stock used or novel genotype generated. Describe any experiments used to assess the effect of a mutation and, where applicable, how potential secondary effects (e.g. second site T-DNA insertions, mosiacism, off-target gene editing) were examined.*
