# Supplementary material for: Author Correction: Morphology shapes community dynamics in early animal ecosystems
Source: Nat Ecol Evol. 2024 Jul 30;8(9):1777. doi: 10.1038/s41559-024-02521-6 (PMC11383793; doi:10.1038/s41559-024-02521-6)
Supplement: Supplementary file 1 — Detailed changes and original figures [file 41559_2024_2521_MOESM1_ESM.pdf]

nature ecology & evolution

Supplementary information

Author Correction: Morphology shapes community dynamics in early animal ecosystems

In the format provided by the authors and unedited

Summary of changes

In our analyses which used our new data, our code did not retrodeform the specimen size data before conducting our tiering analyses. Retrodeformation corrects for the tectonic deformation that occurs within the rocks resulting in stretching of the specimens. The overall fossil areas are the same with and without retrodeformation, so only the tiering analyses are impacted by our mistake. The spatial positions of the fossils were correctly retrodeformed in the original code.

We have since retrodeformed the specimen sizes following the technique of Wood *et al.* 2003 (as originally referenced in the manuscript). There are no qualitative changes to the results reported for tiering in the paper, but we felt a correction should be issued so that the correct tiering statistics and relationships thereof were represented in the study. Generally, after retrodeforming, tiering becomes slightly more prominent, strengthening the relationship between tiering and proportion of stemmed individuals and enhancing the differences between different morphogroups. The relationships between tiering and community succession (W statistic) remains non-significant. The largest changes occur in the old tiering metrics of Mitchell and Kenchington (2018), while our weighted tiering metrics are less impacted.

We have corrected 2 figures and 1 table as part of this correction, and edited the text as explained below. Note that there was a language error in paragraph 1 of the discussion concerning the number of communities that had high, medium, and low levels of tiering that did not match up with statements in the rest of the manuscript.

Detailed description of changes

Main text

In the “Tiering” section, the sentence originally reading “We found that eight communities had high levels of tiering (>0.70), six had medium (0.40–0.70) and four low (<0.40)” has now been corrected to “We found that nine communities had high levels of tiering (>0.70), six had medium (0.40–0.70) and three low (<0.40)”. The sentence “…negatively correlated with weighted DVS^u using Spearman’s rank ($\rho(17) = -0.605$, $P = 0.005$; Fig. 5a)” has been corrected to “…negatively correlated with weighted DVS^u using Spearman’s rank ($\rho(17) = -0.670$, $P = 0.003$; Fig. 5a)”. The text “…strongly positively correlated by Spearman’s rank ($\rho(17) = 0.877$, $P < 0.001$)” has now been corrected to “…strongly positively correlated by Spearman’s rank ($\rho(17) = 0.860$, $P < 0.001$)”. Additionally, “increase of 7%” has replaced “decrease of 0.99%” in the sentence now reading “We found that DVS^u remained the same (mean increase of 7%)”.

Discussion

In the first paragraph of the Discussion, the sentence “…we found that four communities had high levels of tiering (>0.70), six had medium (0.40–0.70) and eight low (<0.40)” has been corrected to “…we found that nine communities had high levels of tiering (>0.70), six had medium (0.40–0.70) and three low (<0.40)”. The text “(mean DVS^h in ref. 21: 0.375 versus mean DVS^h in this study: 0.685)” has been corrected to “(mean DVS^u in ref. 21: 0.375 versus mean DVS^u in this study: 0.647)”. The text “(non-weighted DVS^u: 11% of surfaces highly tiered, 67% not tiered; weighted DVS^u: 44% of surfaces highly tiered, 22% not tiered)” has been corrected to (non-weighted DVS^u: 11% of surfaces highly tiered, 61% not tiered; weighted DVS^u: 50% of surfaces highly tiered, 17% not tiered)”. Additionally, “nine” has replace “eight” in the sentence now reading “Therefore, the change from one (non-weighted) to nine (weighted) highly tiered communities…”.

In the third paragraph of the Discussion, “nine” has replaced “eight” in the sentence now reading “… we found high levels of tiering in nine out of 18 surfaces” and “68.3%” has replaced “54.7%” in the sentence now reading “The prevalence of tiering is greater (DVS^u of 68.3%)”.

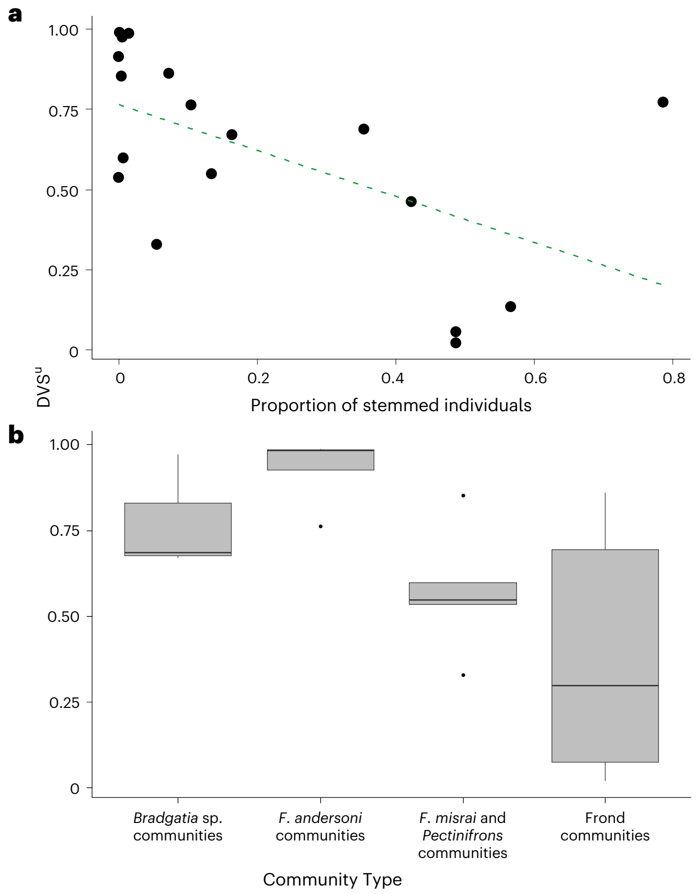

Original Fig. 5

Original Extended Data Table 1

	DVS Statistics				Mean W Statistics			
**Surface**	**DVS^h**	**DVS^u**	**Weighted DVS^h**	**Weighted DVS^u**	**No effaced fronds**	**Effaced fronds as additional taxon**	**Effaced fronds proportionally relabelled**	**'Best guess' applied to effaced fronds where possible**
Bed B	0.065	0.168	0.061	0.134	0.049	0.033	0.048	0.042
Bishop's Cove	0.246	0.246	0.972	0.972	−0.017	−0.074	−0.135	−0.026
Brasier	0.186	0.336	0.801	0.762	0.089	0.057	−0.015	0.045
Bristy Cove	1	0.826	1	0.981	–	–	–	–
Goldmine	0.496	0.747	0.971	0.985	−0.018	−0.096	−0.157	−0.04
Green Point	0.617	0.246	0.782	0.328	0.019	−0.003	−0.076	0
H14	0.361	0.251	0.996	0.988	0.008	0.004	0.001	0.007
H26	0.901	0.505	0.857	0.77	0.016	−0.239	−0.066	0.067
H38	0.515	0.386	0.653	0.67	−0.091	−0.118	−0.195	−0.097
H5	0.058	0.022	0.111	0.02	0.169	0.429	0.274	0.178
LMP	0.161	0.07	0.107	0.056	−0.118	0.186	0.082	−0.023
Melrose	0.612	0.359	0.805	0.535	0.13	0.008	0.007	0.055
D Surface	0.204	0.235	0.854	0.598	0.065	0.019	0.029	0.022
E Surface	0.084	0.1	0.721	0.547	−0.014	−0.013	−0.068	−0.016
G Surface	0.518	0.435	0.563	0.686	−0.056	0.094	−0.002	−0.034
Pizzeria	0.277	0.215	0.385	0.462	0.1	0.137	0.079	0.026
Shingle Head	0.653	0.514	0.923	0.851	–	–	–	–
St. Shott's	0.217	0.425	0.768	0.861	−0.045	−0.036	−0.031	−0.058

W-statistics taken across 1,000 spatial jack-knife subsamples.

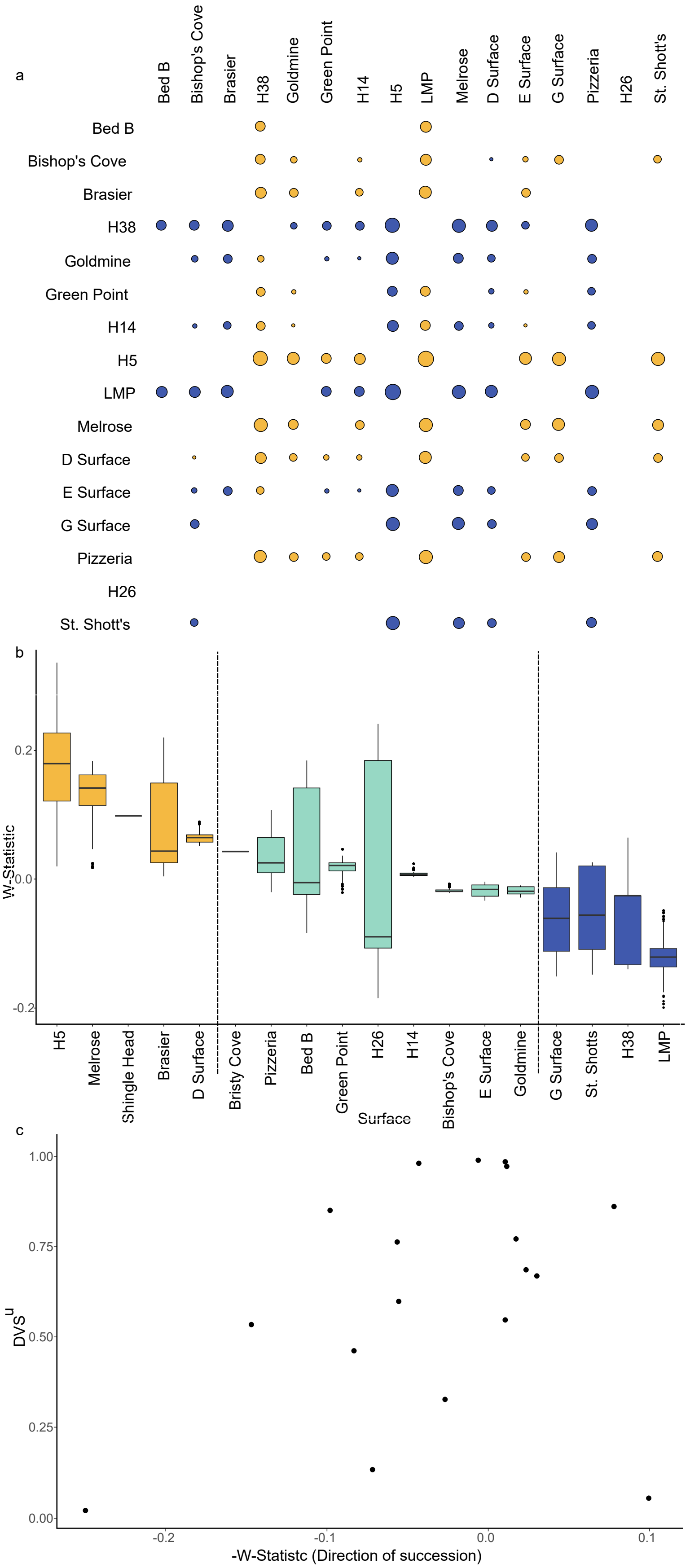

Original Extended Data Fig. 6
